# Is Obesity a Major Driver of Primary Aldosteronism?

**DOI:** 10.1016/j.jacbts.2026.101541

**Published:** 2026-04-27

**Authors:** John E. Hall, Ana C.M. Omoto, Michael E. Hall

**Affiliations:** a Department of Physiology and Biophysics, University of Mississippi Medical Center, Jackson, Mississippi, USA; b Department of Medicine, University of Mississippi Medical Center, Jackson, Mississippi, USA; c Mississippi Center for Obesity Research, University of Mississippi Medical Center, Jackson, Mississippi, USA; d Cardiorenal and Metabolic Diseases Research Center, University of Mississippi Medical Center, Jackson, Mississippi, USA; e Mississippi Center for Clinical and Translational Research, University of Mississippi Medical Center, Jackson, Mississippi, USA

**Keywords:** adipocytes, aldosterone synthase inhibitor, hypertension, idiopathic hyperaldosteronism, mineralocorticoid receptor antagonist, renin

**Figure:**
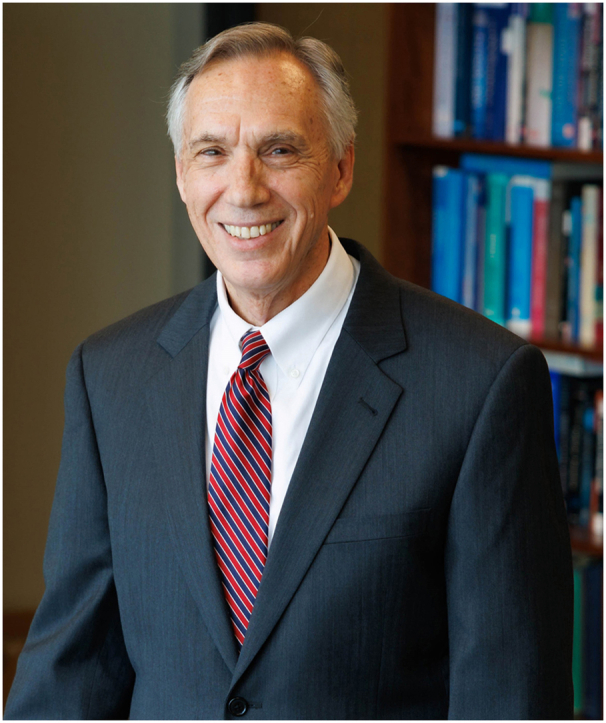

**Figure:**
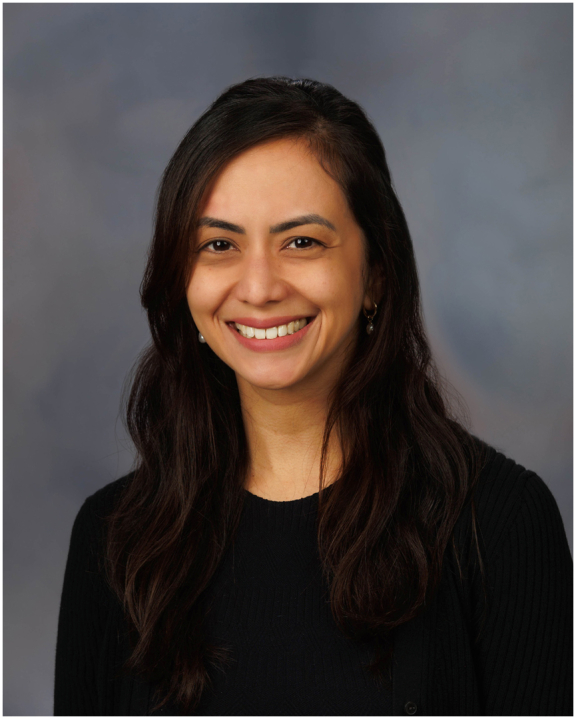

**Figure:**
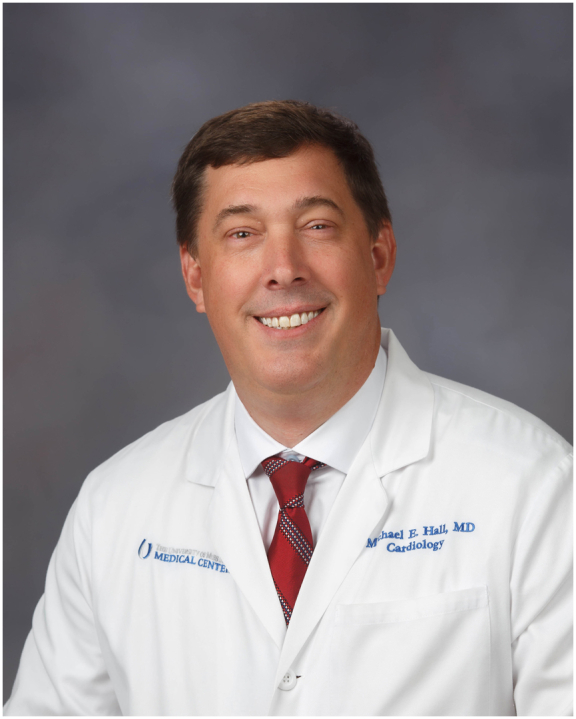

Obesity, especially when associated with excess visceral fat, is widely recognized as a major driver of elevated blood pressure (BP), accounting for 65% to 78% of the risk for primary hypertension.^1^ Although the causes of obesity-associated hypertension are multifactorial, inappropriately elevated plasma aldosterone concentration, relative to sodium balance, is increasingly recognized as a contributor to increased BP, especially in those who have difficult-to-control or resistant hypertension.^2^ Obesity may be a key factor underlying idiopathic hyperaldosteronism, which is characterized by bilateral adrenal hypersecretion of aldosterone. Idiopathic hyperaldosteronism is the most common cause of primary aldosteronism (PA), a group of disorders defined by inappropriately high aldosterone secretion despite suppressed plasma renin activity. Although PA appears to be rising in parallel with increasing rates of obesity, the prevalence of PA is still uncertain and estimates have varied widely, partly due to differences in diagnostic criteria, patient selection, and antihypertensive medications that influence diagnostic measurements (e.g. aldosterone and renin).

In this issue of *JACC: Basic to Translational Science*, Parisien-La Salle et al^3^ provide an extensive and careful phenotyping study of aldosterone and cortisol dysregulation in 77 people with obesity-associated hypertension. Participants taking diuretics, angiotensin-converting enzyme inhibitors, or angiotensin receptor blockers were required to withdraw these drugs for 2 weeks before phenotyping procedures. Using a saline suppression test (SST), an oral sodium loading test, a dexamethasone suppression test (DST), and an adrenocorticotrophic hormone (ACTH) stimulation test to interrogate aldosterone and cortisol regulation, they classified participants into 3 aldosteronism phenotypes: 1) PA phenotype (low renin with nonsuppressible aldosterone); 2) low-renin phenotype (low renin and low aldosterone); and 3) renin-dependent aldosteronism (high renin with renin-mediated aldosteronism). DST was used to assess ACTH-independent hypercortisolism and ACTH stimulation was used to evaluate ACTH-modulated adrenocortical hormone production.

Parisien-La Salle et al observed a much higher prevalence of hyperaldosteronism than previously recognized in people with obesity-associated hypertension. Over 80% of individuals with obesity hypertension in this study had overlapping phenotypes of hyperaldosteronism and/or hypercortisolism. Although 37.7% of the participants had a clear PA phenotype at baseline, the SST “unmasked” a substantial number of people with inappropriately elevated aldosterone despite suppressed renin. Overall, 51.9% of this cohort exhibited the PA phenotype after SST. Individuals with masked PA had overlapping features of PA and renin-dependent aldosteronism and would not have been detected using current recommended screening methods.^4^ Approximately 25% of study participants had persistent renin-dependent hyperaldosteronism that could not be suppressed by sodium loading and 9.2% had ACTH-independent hypercortisolism after DST. The authors concluded that their results support increased emphasis on aldosterone- and cortisol-targeted therapies for obesity-associated hypertension.

A major strength of this study is the extensive phenotyping, which provided insights into the physiology of aldosterone and cortisol dysregulation in obesity-associated hypertension. The extensive phenotyping revealed substantially higher estimates of PA prevalence than previously reported in obese people with mild, well-controlled hypertension; baseline systolic BP prior to testing procedures averaged 128.9 mm Hg and was managed with 0 or 1 antihypertensive agent. Despite notable strengths, this study has a few limitations. Perhaps most important is the relatively small sample size of the cohort, which may limit generalizability of the results. Also, approximately 83% of the people in this cohort were White and only 14% were Black. Another limitation is the lack of a comparator group of nonobese people with hypertension.

Despite these limitations, the results of this study imply that nearly all people with obesity-related hypertension have some manifestation of hyperaldosteronism and/or hypercortisolism and would likely benefit from targeting these abnormalities. Thus, these results support greater use of mineralocorticoid receptor antagonists (MRAs) or the emerging aldosterone synthase inhibitors (ASIs) for treating obesity-associated hypertension. Although MRAs are recommended as a fourth-line agent for treating resistant hypertension in people with a glomerular filtration rate (GFR) >30 mL/min/1.73 m^2^ and plasma potassium <5.0 mEq/L, they are not routinely used for initial therapy of primary hypertension, which is, to a great extent, driven by excess adiposity.

The mechanisms by which excess adiposity leads to hyperaldosteronism are still unclear but may be multifactorial. Obesity, especially when associated with excess visceral, renal sinus, and pararenal fat, leads to mild activation of the renin-angiotensin-aldosterone system through multiple effects, including increased renal sympathetic nerve activity and physical compression of the kidneys.^5^ Renin-angiotensin-aldosterone system activation occurs despite elevated BP and sodium retention, which normally suppress secretion of renin and aldosterone. However, as demonstrated by Parisien-La Salle et al, nearly 25% of people with obesity-associated hypertension continue to exhibit renin-independent hyperaldosteronism, with inappropriately elevated aldosterone after sodium loading and renin suppression. What is the stimulus for increased secretion of aldosterone when two of its major controllers, plasma renin and potassium levels, are either normal or suppressed?

Several adipocyte-derived factors have been suggested to directly stimulate adrenal aldosterone synthesis and secretion (Figure 1). For example, studies in adrenal tissues of humans and rodents have provided evidence that adipocyte-derived leptin may stimulate aldosterone synthesis and secretion by binding to leptin receptors in adrenal glomerulosa cells and increasing CYP11B2 (aldosterone synthase) expression.^6^ Other adipocyte-derived factors, such as interleukin-6, as well as very low-density lipids have also been suggested to stimulate CYP11B2 expression and aldosterone secretion.^6^ However, the quantitative importance of these factors in contributing to increased aldosterone secretion and hypertension in obese humans is still unclear.

**Figure 1:**
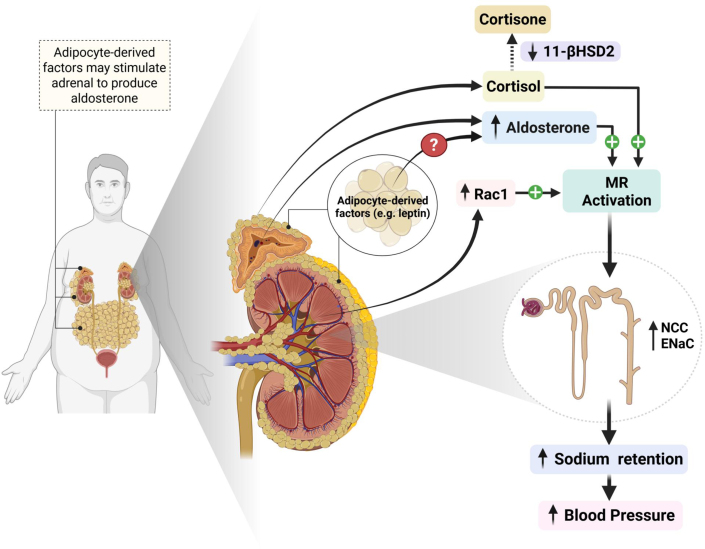
Aldosterone Secretion by Adipocyte-Derived Factors and Aldosterone-Independent MR Activation Potential roles of aldosterone-independent mineralocorticoid receptor (MR) activation and adipocyte-derived factors in stimulating aldosterone secretion. Obesity may enable cortisol-induced MR activation by down-regulating renal tubular 11β-HSD2, which converts cortisol to cortisone. Obesity may also increase renal expression of Rac1, which can activate the MR. Activation of the MR increases renal sodium reabsorption, contributing to extracellular fluid volume expansion and increased blood pressure in obesity. ENaC = epithelia sodium channel; NCC = sodium-chloride co-transporter.

Hypercortisolism may also contribute to activation of the mineralocorticoid receptor (MR) and increased BP in obesity-associated hypertension. In the study by Parisien-La Salle et al, 9.2% of the participants had ACTH-independent hypercortisolism. However, even normal levels of cortisol may activate the MR and raise BP if obesity causes downregulation of 11β-HSD2, as suggested by preclinical studies (Figure 1).^1^ This enzyme is colocalized with the MR in renal collecting tubules and normally converts cortisol to cortisone, which is relatively inactive at the MR.

Obesity also enhances renal tubular expression of Rac1, a small guanosine triphosphate–binding protein member of the Rho family of guanosine triphosphatases that activates MR signal transduction.^7^ Thus, obesity may induce excess renal tubular MR activation even in the absence of elevated aldosterone levels, perhaps explaining why MRAs effectively lower BP in people with obesity and resistant hypertension even when plasma aldosterone levels are normal or reduced after treatment with renin-angiotensin system blockers.^1^

As emphasized by Parisien-La Salle et al, their results should not be interpreted as encouragement for more PA testing, but rather as recognition that most people with obesity-associated hypertension would likely benefit from treatment with MRAs or ASIs. We concur, although there are still many unanswered questions. For example, the mechanisms by which obesity leads to renin-independent hyperaldosteronism are still unclear. Perhaps a more important question, from a clinical perspective, is whether earlier and more widespread use of MRAs, including steroidal (spironolactone and eplerenone) and nonsteroidal options (finerenone), as well as emerging ASIs, would prevent target organ injury and development of resistant hypertension in people with obesity. Another important question is whether there are significant differences in the efficacy and safety of MRAs and ASIs in treating primary hypertension. Major clinical trials indicate that both classes of drugs are effective in reducing BP in treatment-resistant hypertension.^8^^,^^9^ Although most people with resistant hypertension are overweight/obese, a randomized clinical trial with head-to-head comparison of MRAs and ASIs has not, to our knowledge, been conducted. If there is significant aldosterone-independent MR activation in obesity, MRAs may have greater efficacy than ASIs in reducing BP.

Concerns for hyperkalemia and reductions of GFR are important reasons that MRAs are not more widely used for antihypertensive therapy. However, obesity-induced hypertension is often associated with low-normal plasma potassium concentration and glomerular hyperfiltration, which contributes to progressive kidney injury.^1^^,^^5^ Preclinical studies and clinical trials have shown that administration of MRAs markedly attenuates glomerular hyperfiltration and target organ injury in chronic obesity-induced hypertension. Although treatment with MRAs and ASIs requires monitoring of plasma potassium and GFR, earlier use of MRAs and ASIs may prevent development of target organ injury and resistant hypertension in obesity-associated hypertension.

## Funding Support and Author Disclosures

The research was supported by grants from the National Heart, Lung, and Blood Institute (R01 HL181254 and R01 HL163076), National Institute of General Medical Sciences (P30 GM149404, and U54 GM115428), and American Heart Association (25CDA1451524). The content is solely the responsibility of the authors and does not necessarily represent the official views of the National Institutes of Health or the American Heart Association. All other authors have reported that they have no relationships relevant to the contents of this paper to disclose.
